# The atypical chemokine receptor-2 does not alter corneal graft survival but regulates early stage of corneal graft-induced lymphangiogenesis

**DOI:** 10.1007/s00417-018-4070-1

**Published:** 2018-07-27

**Authors:** Tian Yu, J. V. Forrester, Gerard J. Graham, Lucia Kuffova

**Affiliations:** 1 Division of Applied Medicine, Section of Immunity, Infection and Inflammation (Ocular Immunology), Institute of Medical Sciences, School of Medicine, Medical Sciences and Nutrition, University of Aberdeen, Foresterhill, Aberdeen, AB25 2ZD UK; 2 Ocular Immunology Program, Centre for Ophthalmology and Visual Science, The University of Western Australia, Perth, Western Australia 6009 Australia; 3 Centre for Experimental Immunology, Lions Eye Institute, Nedlands, Perth, Western Australia 6009 Australia; 4 Chemokine Research Group, Institute of Infection, Immunity and Inflammation, College of Medical, Veterinary and Life Sciences, University of Glasgow, Glasgow, G12 8TT UK; 5 NHS Grampian, Aberdeen, UK

**Keywords:** ACKR2, Corneal transplantation, Lymphangiogenesis, Angiogenesis, Graft rejection, Chemokines

## Abstract

**Purpose:**

To re-evaluate the role of the atypical chemokine receptor-2 (ACKR2) in corneal graft rejection and investigate the effect of ACKR2 on inflammation-associated lymphangiogenesis using murine orthotopic corneal transplantation.

**Methods:**

Corneal grafts were performed and evaluated in the settings of syngeneic, allogeneic and single antigen (HY-antigen) disparity pairings. Corneal vessels were quantified in whole mounts from WT, ACKR2^−/−^ and F4/80^−/−^ACKR2^−/−^ mice that received syngeneic or allogeneic grafts using anti-CD31 and anti-Lyve-1 antibodies.

**Results:**

Syngeneic corneal grafts in WT and ACKR2^−/−^ mice were 100% accepted. Fully histo-incompatible allogeneic grafts were rapidly rejected (100%) with similar tempo in both WT and ACKR2^−/−^ hosts. Around 50% of single-antigen (HY) disparity grafts rejected at a slow but similar tempo (60 days) in WT and ACKR2^−/−^ mice. Prior to grafting, F4/80^−/−^ACKR2^−/−^ mice had lower baseline levels of limbal blood and lymphatic vessels compared to ACKR2^−/−^ mice. Syngeneic grafts, but not allogeneic grafts, in ACKR2^−/−^ and F4/80^−/−^ACKR2^−/−^ mice induced higher levels of lymphatic sprouting and infiltration of Lyve-1^+^ cells during the early (3d) post-graft (pg) stage but lymphatic density was similar to WT grafted mice by 7d pg.

**Conclusions:**

Our results indicate that the chemokine scavenger receptor, ACKR2, has no role to play in the survival of allogeneic grafts. A minor role in regulation of lymphangiogenesis in the early stage of wound healing in syngeneic grafts is suggested, but this effect is probably masked by the more pronounced lymphangiogenic inflammatory response in allogeneic grafts. No additional effect was observed with the deletion of the resident macrophage gene, F4/80.

## Introduction

Corneal allograft rejection is predominantly mediated through the indirect pathway of allorecognition whereby newly recruited host antigen presenting cells (APC) process and present corneal alloantigens to naïve host T cells. The activation of an allospecific Th1 response then promotes the rejection of corneal allograft [reviewed in [1]]. Unlike allografts, syngeneic corneal grafts performed in naïve hosts are accepted indefinitely [2]. However, despite different outcomes, both corneal syngeneic and allogeneic grafts induce corneal hem- and lymph-angiogenesis which are considered to significantly affect the fate of the graft or the success of a second graft [3, 4].

The atypical chemokine receptor-2 (ACKR2, formerly known as D6) is a chemokine decoy receptor expressed mainly on afferent lymphatic endothelial cells (LEC) and innate-like B cells as well as some other leukocyte subsets such as dendritic cells (DC), macrophages and neutrophils [5] and has also recently been identified on a stromal fibroblastic population in the murine mammary gland [6]. Chemokine decoy receptors, such as ACKR2, have similar structures to conventional chemokine receptors, but they behave anomalously by binding chemokine ligands which fail to initiate G-protein-dependent signalling. Instead, engaged ligands are degraded after internalisation, and, in the case of ACKR2, the receptor is recycled back to the cell surface [5, 7]. ACKR2 recognises most pro-inflammatory CC chemokines with different affinity, but does not recognise homeostatic chemokines [5]. In this way, ACKR2 is regarded as a chemokine scavenger that regulates pro-inflammatory CC chemokine levels which in turn modulate immune responses.

Several studies have shown that ACKR2 is involved in the efficient resolution of inflammation. The absence of ACKR2 leads to more severe inflammatory disease associated with increased chemokine and leukocyte infiltrations [5, 8–10]. Furthermore, in a previous study, deletion of ACKR2 was reported to be associated with significantly increased rejection of corneal syngeneic grafts with nearly 60% of ACKR2^−/−^ mice rejecting their syngrafts at 1 week post-surgery [11]. This is a surprising result since ACKR2^−/−^ mice are fully histo-compatible with wild-type littermate mice and are in effect syngeneic grafts. However, the effect was ascribed to an elevated innate immune response in ACKR2^−/−^ mice which, under sterile conditions of transplant surgery, implies involvement of damage-associated molecular patterns (DAMPS) [12]. Specifically, it was suggested that ACKR2 expressed by DC plays a role in modifying DC behaviour by promoting maturation and allosensitisation [11]. However, despite showing an impaired allospecific T cell response in ACKR2^−/−^ mice, allograft survival was not different between WT and ACKR2^−/−^ mice [11], suggesting that any such effect only affected syngeneic grafts.

An alternative role for ACKR2 function in the innate immune system has recently been suggested. ACKR2 regulates the embryonic development of lymphatic vessels, and that deletion of ACKR2 eventually leads to increased lymphatic vessel density in adult mice in a range of tissues including the skin, diaphragm, and lymph nodes [13]. This phenotype was associated with increased proximity of pro-lymphangiogenic macrophages to LEC in ACKR2^−/−^ mice [13]. However, it is not clear whether ACKR2 is involved in inflammation-associated lymphangiogenesis in adult mice.

In view of the recognised importance of both chemokines [14] and lymphangiogenesis [4] in corneal graft rejection, we have re-investigated the role of ACKR2 in mouse corneal graft survival using ACKR2-deficient mice. In addition, in view of the proposed role of macrophages and DC in this process [11, 13], we have also evaluated corneal neovascularisation in ACKR2:F4/80 double gene deficient mice. F4/80 is expressed predominantly on resident tissue, suppressive macrophages and a subset of DC [15, 16]. We compared syngeneic, fully MHC incompatible allogeneic and single antigen disparity (HY) graft survival in WT and ACKR2^−/−^ mice and evaluated blood and lymphatic corneal vessel growth in all groups of mice with syngeneic or allogeneic corneal grafts. We observed no difference in corneal graft survival between WT and ACKR2^−/−^ in all settings tested while there was only a transient (first 3 days post-graft, [pg]) pro-lymphangiogenic effect on corneal vessels in ACKR2^−/−^ mice. We are therefore unable to confirm a significant role for ACKR2 in the regulation of innate immune responses in the setting of murine corneal graft or in corneal syn- or allo-graft rejection.

## Materials and methods

### Mice

ACKR2^−/−^ (background C57BL/6, H2^b^) and WT (H2^b^) littermates were used for all experiments. F4/80^−/−^ mice (kindly provided by Professor Siamon Gordon, University of Oxford, UK) were crossed with ACKR2^−/−^ mice to produce double knockout F4/80^−/−^ACKR2^−/−^ mice (background C57BL/6, H2^b^). Female Balb/c (H2^d^) mice were used as donors for allogeneic corneal grafts. Syngeneic grafts were performed in WT to WT and ACKR2^−/−^ to ACKR2^−/−^ combinations. All mice were bred and kept at the Medical Research Facility, University of Aberdeen. Sex-matched 6–8-week-old mice were used in all experiments. All animals were treated in accordance with guidelines of the Association for Research in Vision and Ophthalmology (ARVO) Statement for the use of animals in ophthalmic and vision research and the regulations of the Animals (Scientific Procedures) Act 1986.

### Orthotopic corneal transplantation

The mouse full-thickness orthotopic corneal transplantation was performed as described previously [17, 18]. Briefly, donor cornea marked with a 2.0 mm trephine was excised and sutured onto a 1.5-mm graft bed by one continuous suture using 11-0 Ethilon (Ethicon, New Jersey, USA). Grafted corneas were examined and monitored under an operating microscope after the procedure and corneal graft opacity scored as described previously [18]. Corneal graft opacity scores greater than or equal to 2 were considered as rejected grafts [18].

### Corneal whole mount

Preparation of corneal whole mount was performed as previously described with modifications [17, 19]. The corneas including corneal limbus were excised together with the lens and fixed immediately in 4% paraformaldehyde at 4 °C for 30 min. The lens and iris were then removed from the corneas and the corneas were incubated in methanol for 20 min at room temperature. Permeabilization was performed by incubating the corneas in 0.3% Triton X-100 overnight at 4 °C. The corneas were then blocked with 10% normal mouse serum for 30 min at room temperature before incubated with rat anti-mouse CD31 (550274, BD Bioscience) and rabbit anti-mouse Lyve-1 (ab14917, Abcam, UK) antibodies diluted in PBS-BGEN (3% BSA, 0.25% gelatine, 5 mM EDTA and 0.025% IGEPAL CA-630 equivalent to Nonidet-P40) overnight at 4 °C. Directly conjugated secondary antibodies Alexa Fluor 555 goat anti-rat IgG (A21434, Invitrogen) and Alexa Fluor 488 goat anti-rabbit IgG (A11070, Invitrogen) diluted in PBS-BGEN were then incubated with the cornea for 2 h at room temperature. Corneas were washed 5 × 5 min between incubations with PBS. After staining, the corneas were mounted with Hydromount and imaged with Zeiss slide scanner (Zeiss Axio Scan.Z1, Zeiss, Jena, Germany).

### Vessel quantification

Images acquired from corneal whole mounts were then analysed by the Volocity software (PerkinElmer, MA, USA) for areas of vessel coverage. The area of blood and lymphatic vessels was determined as between outer corneal limbal vessel arcade to the innermost end of newly formed vessels. For quantification of lymphangiogenesis, the numbers of sprouts and loops per cornea were quantified in a masked manner using the ImageJ software (National Institute of Health, USA) with the plug-in Lymphatic Vessel Analysis Protocol (LVAP).

### Statistical analysis

GraphPad Prism (GraphPad Software, USA) was used for all statistical analysis. Log-rank test was used for comparison of corneal graft survival. For vessel quantifications, one-way ANOVA was used with Bonferroni post-test analysis. Statistical significance was considered when *p* < 0.05.

## Results

### Survival of murine corneal syngeneic and allogeneic grafts are unaffected by deletion of ACKR2

**Fig. 1:**
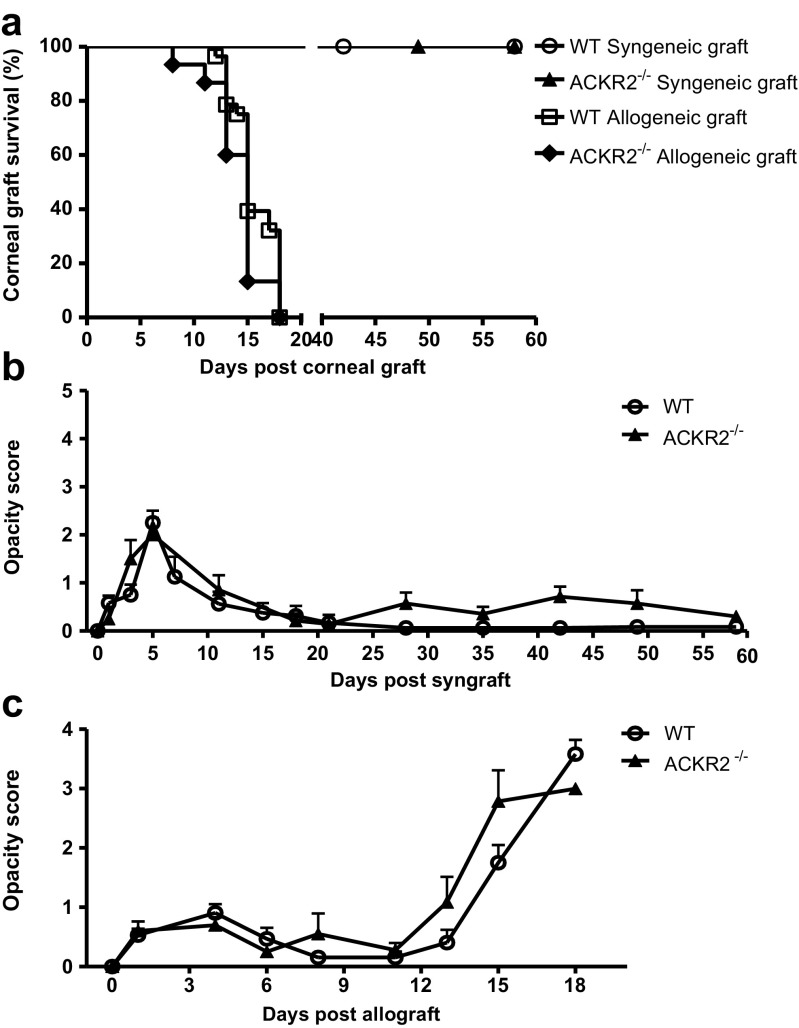
Corneal syngeneic and allogeneic graft survival and opacity scores. WT and ACKR2^−/−^ mice were grafted with sex-matched syngeneic or allogeneic (Balb/c) corneal grafts, and corneal opacity was scored at intervals post-surgery. (**a**) Corneal graft survival in WT and ACKR2^−/−^ mice. Corneal opacity scores of syngeneic grafts (**b**) and allogeneic grafts (**c**). Syngeneic grafts: n^WT^ = 8, n^ACKR2−/−^ = 7; allogeneic grafts: n^WT^ = 15, n^ACKR2−/−^ = 28. Statistical analysis was performed using Log-rank test for graft survivals and one-way analysis of variance (ANOVA) for corneal opacity scores

Syngeneic corneal grafts in naïve WT mice survive indefinitely [2, 20]. To confirm that deletion of ACKR2 alters graft survival in mice, we grafted sex-matched respective WT and ACKR2^−/−^ donor corneas. No difference in graft survival or opacity score was observed in any combination of syngeneic graft for the duration of the experiment (60 days) (Fig. 1a). Furthermore, corneal graft opacity scores demonstrated that both WT and ACKR2^−/−^ mice experienced similar levels of transient corneal opacification during the first week post-syngraft (Fig. 1b). This transient effect is known to be due to infiltration of innate immune cells caused by the surgery and by temporary distress of corneal endothelial cells [17, 18]. By the third week, all syngrafts were clear (Fig. 1b).

Fully mismatched, donor corneal allografts (Balb/c, H2^d^) to WT (C57BL/6, H2^b^) and ACKR2^−/−^ recipients (H2^b^) were rapidly rejected with peak of rejection incidence occurring at day 13–18 pg and corneal opacity scores reaching 3 to 4 (Fig. 1a, c).

### Rejection rate of single antigen (HY-antigen) disparity corneal grafts is not different between WT and ACKR2^−/−^ mice

**Fig. 2:**
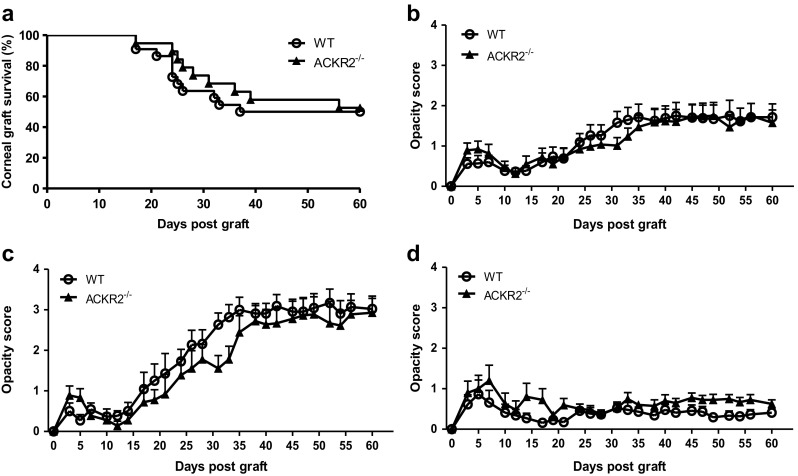
Corneal graft survival and opacity scores of HY-antigen disparity group. Female WT and ACKR2^−/−^ mice were grafted with strain-matched male donor corneas and corneal opacity was scored at intervals post-surgery. (**a**) Corneal graft survival of HY-antigen disparity grafts in WT and ACKR2^−/−^ mice (n^WT^ = 22, n^ACKR2−/−^ = 19). (**b**) Overall corneal opacity scores of WT and ACKR2^−/−^ mice receiving HY-antigen disparity donor grafts (n^WT^ = 22, n^ACKR2−/−^ = 19). Corneal grafts were subdivided into (**c**) “rejectors” (n^WT^ = 11, n^ACKR2−/−^ = 9) and (**d**) “acceptors” (n^WT^ = 11, n^ACKR2−/−^ = 10) and corneal opacity scores were compared between WT and ACKR2^−/−^ mice. Statistical analysis was performed using Log-rank test for corneal graft survival and one-way ANOVA for corneal opacity scores

Fully mismatched corneal allograft induces strong innate and adaptive immune responses involving inflammatory chemokines [14]. ACKR2 has been shown to down-regulate the inflammatory response and contribute to the resolution of inflammation by clearance of inflammatory chemokines in various inflammatory models [5], in part by facilitating lymph flow and migration of APC during inflammation [21, 22]. The lack of effect of ACKR2 in corneal syngeneic and allogeneic graft rejection was therefore surprising. However, it is possible that the strength of the alloreaction in fully mismatched corneal allograft has overwhelmed any possible effect of ACKR2 in chemokine regulation. Therefore, we evaluated graft survival in a single disparity, non-MHC pairing (HY-antigen) group by grafting male donor corneas to female recipients, comparing M-F WT grafts with M-F ACKR2^−/−^ grafts on the C57BL/6 background. In this experiment, a milder rejection tempo was observed with graft rejection commencing 2 weeks post-surgery and approximately 50% of grafts rejected by 60d pg (Fig. 2a). However, there was no difference in corneal opacity or graft survival rate between WT and ACKR2^−/−^ mice (Fig. 2a, b). As the corneal opacity scores, as well as the immunological status of recipient mice vary significantly between mice that accepted or rejected corneal grafts, the recipient mice were grouped into “rejectors” and “acceptors” according to corneal graft outcomes by day 60 pg and corneal opacity scores were re-analysed. When “rejectors” only were evaluated, the tempo of rejection was slightly increased in single disparity grafts but the absence of AKCR2 did not alter the rejection rate (Fig. 2c, d). Therefore, these findings indicate that ACKR2 does not play a significant role in the rejection or survival of corneal grafts.

### Absence of ACKR2 leads to accelerated corneal lymphangiogenesis

**Fig. 3:**
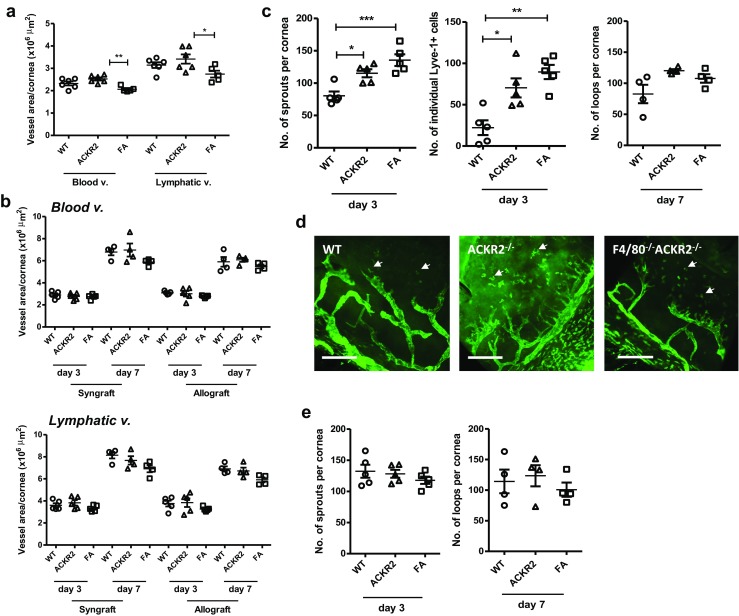
Quantification of normal corneal limbal vascularisation and neovascularisation post corneal graft. Corneal whole mounts were prepared from naïve or grafted (syngeneic and allogeneic) WT, ACKR2^−/−^ (ACKR2), F4/80^−/-^ACKR2^−/−^ (FA) mice and stained with anti-CD31 and anti-Lyve-1 antibodies for blood and lymphatic vessels, respectively. Corneal vascularisation was quantified by area covered by vessels (**a**, **b**) and corneal lymphangiogenesis was further assessed by lymphatic sprouts, loops and corneal infiltration of individual Lyve-1^+^ cells (**c**, **e**). (**a**) Quantification of normal corneal limbal blood and lymphatic vessels (n^WT^/n^ACKR2−/−^ = 6, n^F4/80−/−ACKR2−/−^ = 5). (**b**) Quantification of blood and lymphatic vessels at day 3 (*n* = 5) and day 7 (*n* = 4) post-corneal syngeneic and allogeneic graft. (**c**) Evaluation of corneal lymphangiogenesis in syngeneic corneal grafts by lymphatic sprouts, corneal infiltration of individual Lyve-1^+^ cells and lymphatic loops formation (n^day3^ = 5, n^day7^ = 4). (**d**) Representative images of corneal syngeneic graft at day 3 pg stained with anti-Lyve-1 antibody. Corneal infiltration of individual Lyve-1^+^ cells is indicated by white arrows. Scale bar represents 250 μm. (**e**) Evaluation of lymphangiogenesis in corneal allografts at day 3 (*n* = 5) and day 7 pg (*n* = 4). Statistical significance was determined using one-way ANOVA, *p < 0.05, **p < 0.01, ***p < 0.001

Corneal graft rejection is associated with significant corneal vascularisation, including lymphangiogenesis [23]. ACKR2 is involved in regulating the establishment of lymphatic vessels during embryonic development mediated in part by pro-lymphangiogenic macrophages residing close to the developing lymphatics [13]. We sought to investigate whether ACKR2 is also required for regulating corneal inflammation-associated lymphangiogenesis during corneal graft. The cornea is normally avascular with vessels present only at the peripheral corneal limbus. Inflammation-associated corneal neovascularisation leads to the invasion of blood and lymphatic vessels towards the centre of cornea. Furthermore, macrophages play important roles in promoting corneal inflammation-associated lymphangiogenesis and studies have reported that the macrophage surface protein, F4/80, is required for ensuring the basal resting limbal lymphatic vessel numbers [24]. Lack of F4/80 has also been shown to be associated with a significantly reduced level of lymphangiogenesis in a model of corneal suture-induced neovascularisation [24]. Therefore, to investigate the role of ACKR2 and its association with macrophages in corneal lymphangiogenesis, quantification of corneal neovascularisation after corneal syngraft and allograft was performed in WT, ACKR2^−/−^ and F4/80^−/-^ACKR2^−/−^ mice. First, the baseline amount of limbal vessels in normal, non-transplanted mice was quantified by corneal whole mount imaging of CD31 and Lyve-1 staining and analysed by the Volocity software for area covered by blood and lymphatic vessels, respectively (Fig. 3a). Our data show that the levels of WT and ACRR2^−/−^ corneal limbal vessels in the unchallenged eye were similar, whereas F4/80^−/−^ACKR2^−/−^ mice had reduced levels of both blood (*p* < 0.01) and lymphatic vessels (*p* < 0.05) (Fig. 3a). Corneal graft induced marked vascularisation as expected but no difference in either blood or lymphatic vessel growth (measured by area at d3 and d7) was observed in any of the mice (Fig. 3b).

The process of lymphangiogenesis progresses through specific morphological stages including development of initial lymphatic sprouts, formation of lymphatic network/loops and finally maturation and remodelling of lymphatic vessels [25]. We therefore wished to determine whether ACKR2 or F4/80 had any effect on these processes during corneal graft rejection. We found that ACKR2^−/−^ mice developed greater numbers of sprouts compared to WT mice at 3 days post-syngeneic corneal graft (*p* < 0.05; Fig. 3c, d) and a similar trend between WT and F4/80^−/−^ACKR2^−/−^ was observed (*p* < 0.001; Fig. 3c, d). However, no additional effect was seen in ACKR2^−/−^ mice with co-deletion of F4/80 (Fig. 3c, d). Interestingly, the numbers of single Lyve-1^+^ cells (Fig. 3d, arrow) in the corneas followed the same trend in syngrafts at 3d pg in that both ACKR2^−/−^ (*p* < 0.05) and F4/80^−/−^ACKR2^−/−^ mice (*p* < 0.01) showed increased infiltration of these cells compared to WT mice (Fig. 3c, d). These differences were not recapitulated in mice with syngeneic grafts at 7d pg as suggested by similar number of loops between WT, ACKR2^−/−^ and F4/80^−/-^ACKR2^−/−^ mice (Fig. 3c). Furthermore, no difference in lymphangiogenic sprouts or loops was observed in allogeneic grafts at any time point between WT and ACKR2^−/−^ or F4/80^−/−^ACKR2^−/−^ mice (Fig. 3e). Thus, in the syngrafted ACKR2^−/−^ and F4/80^−/-^ACKR2^−/−^ mice, we have observed an accelerated initial lymphatic sprouting response together with increased abundance of Lyve-1^+^ individual cells. However, this did not lead to an increase in overall lymphatic vessel density.

## Discussion

The role of ACKR2 in various inflammatory models is considered to be a general one of limiting excessive inflammation and promoting inflammatory resolution by “scavenging” inflammatory chemokines [5, 8–10]. Few studies have addressed the role of ACKR2 in allo-immunity [11, 26, 27]. Deletion of ACKR2 had a protective effect in graft-versus-host disease attributed to increased numbers and enhanced immunosuppressive activity of Ly6C^high^ monocytes [26] while a pro-inflammatory effect was observed in syngeneic but not allogeneic corneal grafts [11]. It was further proposed in this study that ACKR2 promoted DC maturation and T cell activation [11]. Thus, ACKR2 appears to promote innate immunity and in its absence the tempo of corneal allograft rejection might be expected to be reduced, while syngeneic grafts performed under sterile conditions should not be rejected. Counter-intuitively, syngeneic corneal grafts, which are universally accepted in uninfected mice [2, 18, 20] were reported to be rejected in ACKR2^−/−^ mice while allogeneic corneal graft showed no difference in rejection rates between in WT and ACKR2^−/−^ mice [11].

In view of the importance of chemokines to the corneal graft rejection process, not least because of possible translational and therapeutic implications, we considered it important to revisit the role of ACKR2 in corneal graft rejection. Using three different donor and recipient combinations (syngeneic, allogeneic and HY-antigen disparity), our data report no difference in corneal graft survival between WT and ACKR2^−/−^ mice (Figs. 1 and 2). Importantly, in the previous study, C57BL/6 WT donor corneas were used for both WT and ACKR2^−/−^ mice in syngeneic corneal grafts thus introducing an antigen disparity which may have affected their results [11]. However, the graft rejection rate did not follow the pattern of a single antigen disparity (see Fig. 2 herein) but was rather that of primary endothelial cell failure [18]. Differences in technique may explain the difference between the results of the present study and those of Hajrasouliha et al. [11]. No information is provided on the use of littermate controls in the previous study [11]. In addition, corneal grafts performed using interrupted sutures, as used in the previous study [11], cause significantly greater trauma and subsequent corneal opacity at week 1 pg compared to a continuous suture technique used here [18] and lead to greater inflammation and subsequent stress especially to the corneal endothelial cells [18]. Such technical issues will have a significant impact on innate immune activation.

Although we observed no difference in corneal graft rejection rates linked to ACKR2, the possibility that the process of lymphangiogenesis was altered could not be excluded, particularly since such changes had been observed in embryonic skin tissues in AKCR2^−/−^ mice [13]. Overall, the level of peri-corneal limbal lymphatic vessels in resting ACKR2^−/−^ mice was not different from naïve WT mice (Fig. 3a). This finding was not unexpected as the naïve corneal tissues are avascular with a narrow circumferential ring of limbal blood and lymphatic vessels. Whereas shown in the previous study, deletion of ACKR2 altered the density of lymphatic vessel network [13]. However, in agreement with a previous study in F4/80^−/−^ mice [24], we observed that the levels of blood and lymphatic vessels at the corneal limbus in naïve F4/80^−/−^ACKR2^−/−^ mice were significantly reduced compared to WT and ACKR2^−/−^ mice (Fig. 3a). This suggests that F4/80^+^ macrophages are likely required for the development of normal corneal limbal vessels. Further support for this concept has been shown in the embryonic skin of WT and ACKR2^−/−^ mice where two distinct populations of macrophages were identified namely CD11b^hi^F4/80^lo^Lyve-1^−^ and CD11b^lo^F4/80^hi^Lyve-1^+^ with the latter population expressing higher pro-angiogenic transcripts [13]. Moreover, previously reported experiments of corneal suture-induced lymphangiogenesis also revealed significant suppression of lymphangiogenesis in F4/80^−/−^ mice compared to WT mice implicating an important role of F4/80^+^ macrophages in corneal lymphangiogenesis [24]. In contrast, we found that after corneal syngeneic graft, F4/80^−/−^ACKR2^−/−^ mice had increased numbers of lymphatic sprouts and increased infiltration of single corneal Lyve-1^+^ cells compared to WT mice (Fig. 3c). Thus, our data suggest that the absence of AKCR2 promoted the recruitment of Lyve-1^+^F4/80^−^ cells which may be capable of promoting the initial lymphatic sprouting and rescued the phenotype of impaired lymphangiogenesis in F4/80^−/−^ mice as reported before [24]. The process of postnatal lymphangiogenesis is known to involve the proliferation and differentiation of LEC after activation by pro-lymphangiogenic stimuli (e.g., vascular endothelial growth factors) and resulting in the sprouting of existing lymphatics [28]. However, increasing evidence supports the notion that bone marrow-derived lymphatic endothelial progenitor cells might differentiate into LEC and contribute to postnatal de novo lymphovasculogenesis in certain tissues during inflammatory response [23, 29–31]. Moreover, these cells may also play a paracrine role in promoting lymphangiogenesis by producing pro-lymphangiogenic cytokines [30]. The effect of ACKR2 and F4/80 was transient and restricted to the early stages of injury since no differences were observed at 7d pg in syngeneic grafts (Fig. 3c). It has been previously shown that syngeneic and allogeneic corneal grafts displayed similarly increased levels of chemokines as well as inflammatory cytokines at early time points (within 1 week pg) [32]. Thereafter, chemokine and cytokine levels remained high in the allogeneic group only, whereas in the syngeneic group, these levels declined significantly [32]. Furthermore, our findings coincide with a recent report that deletion of ACKR2 led to accelerated development of mammary gland branching associated with macrophage recruitment [6]. It was further shown that ACKR2 is differentially expressed in the mammary gland during different biological stages with maximum expression correlating with increased branching and macrophage recruitment in ACKR2^−/−^ mice [6]. In addition, in vitro experiments demonstrated up-regulation of ACKR2 expression upon exposure to IFN-γ and IL-6 [22]. Therefore, ACKR2 in the current model is likely to function by regulating levels of inflammatory chemokines which in turn affects the recruitment of pro-lymphangiogenic Lyve-1^+^ cells during early time points (3d pg) after syngeneic grafts rather than allogeneic grafts. However at later time points where chemokine levels fall significantly in WT mice, ACKR2 may be less effective. Thus, together with our data, suggests that while ACKR2 may exert variable function during different stages of inflammation, we show here that ACKR2 plays a specific regulatory role of early inflammation-associated lymphangiogenesis in adult mouse.

Interestingly, this effect of ACKR2 on lymphangiogenesis was observed in early stages of inflammation of syngeneic grafts but not allogeneic grafts (Fig. 3e). Since the alloimmune response is more prominent and long lasting compared to the immune response to syngeneic grafts (which is, in effect, similar to an autologous corneal wounding response), we suggest that the strength of alloimmune response may mask a more subtle effect on simple wound healing responses. Alternatively, there may be an early suppression of lymph vessel sprouting mediated by the adaptive alloimmune response. This may merit further investigations.

In summary, we report that in contrast to a previous study [11], ACKR2 does not have a role to play in regulating corneal allograft responses. There is a minor effect mediated by ACKR2 during the early stages of lymphangiogenesis in corneal wound healing type responses, but this effect does not change final outcome of the transplanted corneal grafts.

## References

[1] Yu T, Rajendran V, Griffith M, Forrester JV, Kuffova L (2016). High-risk corneal allografts: a therapeutic challenge. World J Transplant.

[2] Sonoda Y, Streilein JW (1992). Orthotopic corneal transplantation in mice--evidence that the immunogenetic rules of rejection do not apply. Transplantation.

[3] Dohlman TH, Omoto M, Hua J, Stevenson W, Lee SM, Chauhan SK, Dana R (2015). VEGF-trap aflibercept significantly improves long-term graft survival in high-risk corneal transplantation. Transplantation.

[4] Dietrich T, Bock F, Yuen D, Hos D, Bachmann BO, Zahn G, Wiegand S, Chen L, Cursiefen C (2010). Cutting edge: lymphatic vessels, not blood vessels, primarily mediate immune rejections after transplantation. J Immunol.

[5] Nibbs RJ, Graham GJ (2013). Immune regulation by atypical chemokine receptors. Nat Rev Immunol.

[6] Wilson GJ, Hewit KD, Pallas KJ, Cairney CJ, Lee KM, Hansell CA, Stein T, Graham GJ (2016) Atypical chemokine receptor ACKR2 controls branching morphogenesis in the developing mammary gland. Development. 10.1242/dev.139733 10.1242/dev.139733 PMC5278629 27888192

[7] Weber M, Blair E, Simpson CV, O'Hara M, Blackburn PE, Rot A, Graham GJ, Nibbs RJ (2004). The chemokine receptor D6 constitutively traffics to and from the cell surface to internalize and degrade chemokines. Mol Biol Cell.

[8] Jamieson T, Cook DN, Nibbs RJ, Rot A, Nixon C, McLean P, Alcami A, Lira SA, Wiekowski M, Graham GJ (2005). The chemokine receptor D6 limits the inflammatory response in vivo. Nat Immunol.

[9] Martinez de la Torre Y, Locati M, Buracchi C, Dupor J, Cook DN, Bonecchi R, Nebuloni M, Rukavina D, Vago L, Vecchi A, Lira SA, Mantovani A (2005). Increased inflammation in mice deficient for the chemokine decoy receptor D6. Eur J Immunol.

[10] Di Liberto D, Locati M, Caccamo N, Vecchi A, Meraviglia S, Salerno A, Sireci G, Nebuloni M, Caceres N, Cardona PJ, Dieli F, Mantovani A (2008). Role of the chemokine decoy receptor D6 in balancing inflammation, immune activation, and antimicrobial resistance in Mycobacterium tuberculosis infection. J Exp Med.

[11] Hajrasouliha AR, Sadrai Z, Lee HK, Chauhan SK, Dana R (2013). Expression of the chemokine decoy receptor D6 mediates dendritic cell function and promotes corneal allograft rejection. Mol Vis.

[12] Todd JL, Palmer SM (2017). Danger signals in regulating the immune response to solid organ transplantation. J Clin Invest.

[13] Lee KM, Danuser R, Stein JV, Graham D, Nibbs RJ, Graham GJ (2014). The chemokine receptors ACKR2 and CCR2 reciprocally regulate lymphatic vessel density. EMBO J.

[14] Wallace GR, John Curnow S, Wloka K, Salmon M, Murray PI (2004). The role of chemokines and their receptors in ocular disease. Prog Retin Eye Res.

[15] Lin HH, Faunce DE, Stacey M, Terajewicz A, Nakamura T, Zhang-Hoover J, Kerley M, Mucenski ML, Gordon S, Stein-Streilein J (2005). The macrophage F4/80 receptor is required for the induction of antigen-specific efferent regulatory T cells in peripheral tolerance. J Exp Med.

[16] Gordon S, Hamann J, Lin HH, Stacey M (2011). F4/80 and the related adhesion-GPCRs. Eur J Immunol.

[17] Kuffova L, Netukova M, Duncan L, Porter A, Stockinger B, Forrester JV (2008). Cross presentation of antigen on MHC class II via the draining lymph node after corneal transplantation in mice. J Immunol.

[18] Plskova J, Kuffova L, Holan V, Filipec M, Forrester JV (2002). Evaluation of corneal graft rejection in a mouse model. Br J Ophthalmol.

[19] Nakao S, Zandi S, Faez S, Kohno R, Hafezi-Moghadam A (2012). Discontinuous LYVE-1 expression in corneal limbal lymphatics: dual function as microvalves and immunological hot spots. FASEB journal : official publication of the Federation of American Societies for Experimental Biology.

[20] Yamada J, Streilein JW (1998). Fate of orthotopic corneal allografts in C57BL/6 mice. Transpl Immunol.

[21] Lee KM, McKimmie CS, Gilchrist DS, Pallas KJ, Nibbs RJ, Garside P, McDonald V, Jenkins C, Ransohoff R, Liu L, Milling S, Cerovic V, Graham GJ (2011). D6 facilitates cellular migration and fluid flow to lymph nodes by suppressing lymphatic congestion. Blood.

[22] McKimmie CS, Singh MD, Hewit K, Lopez-Franco O, Le Brocq M, Rose-John S, Lee KM, Baker AH, Wheat R, Blackbourn DJ, Nibbs RJ, Graham GJ (2013). An analysis of the function and expression of D6 on lymphatic endothelial cells. Blood.

[23] Maruyama K, Ii M, Cursiefen C, Jackson DG, Keino H, Tomita M, Van Rooijen N, Takenaka H, D'Amore PA, Stein-Streilein J, Losordo DW, Streilein JW (2005). Inflammation-induced lymphangiogenesis in the cornea arises from CD11b-positive macrophages. J Clin Invest.

[24] Maruyama K, Nakazawa T, Cursiefen C, Maruyama Y, Van Rooijen N, D'Amore PA, Kinoshita S (2012). The maintenance of lymphatic vessels in the cornea is dependent on the presence of macrophages. Invest Ophthalmol Vis Sci.

[25] Tammela T, Alitalo K (2010). Lymphangiogenesis: molecular mechanisms and future promise. Cell.

[26] Savino B, Castor MG, Caronni N, Sarukhan A, Anselmo A, Buracchi C, Benvenuti F, Pinho V, Teixeira MM, Mantovani A, Locati M, Bonecchi R (2012). Control of murine Ly6C(high) monocyte traffic and immunosuppressive activities by atypical chemokine receptor D6. Blood.

[27] Choi W, Byun YJ, Jung E, Noh H, Hajrasouliha AR, Sadrai Z, Chang E, Lee JH, Lee HK (2015). Chemokine decoy receptor D6 mimicking trap (D6MT) prevents allosensitization and immune rejection in murine corneal allograft model. J Leukoc Biol.

[28] Kim H, Kataru RP, Koh GY (2014). Inflammation-associated lymphangiogenesis: a double-edged sword?. J Clin Invest.

[29] Kerjaschki D, Huttary N, Raab I, Regele H, Bojarski-Nagy K, Bartel G, Krober SM, Greinix H, Rosenmaier A, Karlhofer F, Wick N, Mazal PR (2006). Lymphatic endothelial progenitor cells contribute to de novo lymphangiogenesis in human renal transplants. Nat Med.

[30] Lee JY, Park C, Cho YP, Lee E, Kim H, Kim P, Yun SH, Yoon YS (2010). Podoplanin-expressing cells derived from bone marrow play a crucial role in postnatal lymphatic neovascularization. Circulation.

[31] Hall KL, Volk-Draper LD, Flister MJ, Ran S (2012). New model of macrophage acquisition of the lymphatic endothelial phenotype. PLoS One.

[32] King WJ, Comer RM, Hudde T, Larkin DF, George AJ (2000). Cytokine and chemokine expression kinetics after corneal transplantation. Transplantation.

